# Arabidopsides as Signatory Biomarkers of the *Arabidopsis thaliana* Response to Lipopolysaccharides—Metabolomics Insights

**DOI:** 10.3390/ijms27041719

**Published:** 2026-02-10

**Authors:** Ian A. Dubery

**Affiliations:** Research Centre for Plant Metabolomics, Department of Biochemistry, University of Johannesburg, Auckland Park 2006, South Africa; idubery@uj.ac.za

**Keywords:** *Arabidopsis thaliana*, arabidopsides, lipopolysaccharides, microbe-associated molecular patterns (MAMPs), induced resistance, oxylipins, specialized metabolites

## Abstract

The in vivo production of specialized metabolites in response to external stimuli can be genus- and species-specific and involves the activation of linked metabolic pathways. In *Arabidopsis thaliana*, oxidized galactolipids containing oxo-phytodienoic acid (OPDA), known as Arabidopsides, are functionally linked to multiple plant stress events. It has been proposed that Arabidopsides may fulfill the function of storage metabolites of the esterified OPDA moieties to enable early induction and sustained activation of defense-related pathways linked to jasmonic acid (JA). Differential profiles or signatures of the accumulated Arabidopsides result from early utilization or further conjugation and interconversion reactions. Arabidopsides were previously annotated as discriminant metabolites in untargeted metabolomics datasets of extracts of *A. thaliana* leaves infiltrated with lipopolysaccharide (LPS) chemotypes from two pathogens, as well as a non-pathogen. This elicitation response suggests a functional relationship between LPS as a ‘non-self’ defense-triggering stimulus and the synthesis and accumulation of the annotated Arabidopsides. A defense response or priming effect via OPDA release and subsequent JA actions would be particularly advantageous, as it would equip susceptible plants with a more responsive and effective defensive metabolome through metabolic reprogramming. Arabidopsides may thus be regarded as signature metabolites in underscoring the dynamic and condition-dependent nature of microbe-mediated metabolic reprogramming that involves perception of bacterial LPSs from potential pathogens or beneficial rhizobacteria.

## 1. Introduction

Metabolomics is generally regarded as a data-driven, hypothesis-generating discipline or investigative approach. Metabolites are the end products of cellular processes and metabolomics thus bridges the gap between the upstream molecular ‘omics’ data and the eventual phenotype [1,2]. Cellular metabolomes are therefore highly sensitive and responsive to genetic and environmental changes [3]. The primary goal of metabolomics is to identify metabolic patterns and perturbations within biological systems that can assist in the formulation of new hypotheses on biological function(s), such as stress-related adaptive or coping mechanisms related to plant defense. Untargeted metabolomics, in particular, is a global, unbiased analysis that annotates as many small-molecule metabolites as possible without a strong a priori hypothesis. This discovery approach generates large, complex datasets that are mined for potential biomarkers or altered pathways, leading to the formation of new hypotheses [1].

Arabidopsides are known secondary (or specialized) metabolites of *Arabidopsis thaliana* and are responsive to biotic and abiotic stresses [4,5] (details provided in Section 6). Previous untargeted metabolomic investigations from our laboratory identified Arabidopsides as signatory biomarkers of the lipopolysaccharide (LPS)-triggered elicitation of defense responses in *A. thaliana*. Unfortunately, there is a knowledge gap regarding the relationship between inducing agents/stress factors, activation of metabolic pathways, and processes involved in the synthesis of Arabidopsides. Here, the aim is to interpret and understand the accumulation of Arabidopsides in response to microbe-derived LPSs from a systems biology perspective.

## 2. Chemical Characteristics and Functional Properties of Plant Cell and Chloroplast Membranes

Of the three major lipid classes found in plant plasma membranes (PPMs), glycerolipids, sphingolipids, and sterols, the most abundant are glycerolipids (containing a glycerol backbone), comprising phospholipids, galactolipids, triacylglycerols, and sulfolipids [6,7,8]. The composition of the PPM lipids can be modified by structural changes (e.g., acyl chain lengths and the number and position of double bonds), as well as by developmental and environmental factors (e.g., stress-induced oxidation) [9,10]. In addition to phosphatidylglycerol, the major constituents of photosynthetic membranes of chloroplasts are the galactolipids, containing two classes, mono- and digalactosyl diacylglyceride (M/DGDG). The lipid composition of chloroplast membranes varies, with galactolipids increasing in abundance from the outer to the inner envelope and then to the thylakoid membranes.

The plasma membrane also plays a key role in the perception of environmental signals and functions in the recognition of microbe-associated molecular patterns (MAMPs) through surface-located, membrane-spanning pattern recognition receptors (PRRs) [11]. Lipids and lipid-derived molecules may play a role in membrane-localized perception events and are sensitive (as mediators, modulators, or facilitators) to early defense-related processes leading to the onset of resistance to disease [12,13,14]. Post-biosynthetic modifications or remodeling of the involved lipids can play important roles in determining the physicochemical properties of cellular membranes and their associated functions [15]. Such an example is the oxidation of fatty acids found in membrane lipids under stress conditions. These mobilized oxylipins cover a diverse group of oxygenated lipid molecules [16,17,18], including the galactolipids known as Arabidopsides.

## 3. Bacterial Lipopolysaccharides as Membrane-Derived MAMPs

The outer membrane of gram-negative bacteria contains LPSs that fulfill a protective barrier function. As amphipathic glycoconjugate molecules, these lipoglycans can also act as virulence factors and elicitors (or MAMPs) of cellular responses associated with innate immunity. LPSs from bacterial pathogens contain conserved structures capable of triggering defense/immune responses in the host [19]. These include the influx of calcium ions, extracellular alkalinization, a perception-related oxidative burst with production of reactive oxygen and reactive nitrogen species (ROS and NO), reversible protein phosphorylation, mitogen-activated protein kinase (MAPK) activity, expression of defense genes encoding pathogenesis-related (PR) proteins [20,21,22], and induced resistance to varied bacteria [23].

The fundamental mechanism of how LPSs induce defense responses in plants is still unclear. The hydrophobic lipid A moiety (a phosphorylated disaccharide of glucosamine decorated with multiple fatty acid acyl chains that embed it into the membrane) has been identified a MAMP-active determinant of LPSs. However, subsequent studies have reported an additional role for the hydrophilic core oligosaccharide (COS) and repeat units within the O-polysaccharide moieties (OPS) [22,24]. It is probable that all three components of intact LPS may exhibit distinct MAMP activities [22,25]. In addition, it is yet to be resolved if the unknown LPS receptor(s) involve co-receptors or accessory proteins. An alternatively spliced LPS-responsive lectin (S-domain) receptor-like kinase (RLK) was reported to be involved in LPS perception [26].

LPS can induce localized and systemic resistance responses in plant tissues similar to induced systemic resistance (ISR), protecting them from future pathogen attacks [23]. While metabolic stress responses in *A. thaliana* triggered by LPS have been reported, the induced adaptive responses involve a number of different metabolic pathways (e.g., phenylpropanoid and glucosinolate pathways) that are seemingly responsive to both salicylic acid (SA) as well as jasmonic acid (JA) regulation [27]. Although newly synthesized metabolites have been identified to be associated with the induced responses, no single class of specialized metabolites have yet been proposed as discriminant biomarkers based on metabolomics data.

## 4. Occurrence of Arabidopsides

While galactolipids are regarded as the most abundant lipids on earth, the esterified and oxidized galactolipids, known as Arabidopsides, belong to a class of specialized metabolites first identified in *A. thaliana* [4,5] (details provided in Section 6). These molecules were discovered in extracts from leaves, but Arabidopsides have also been reported in other tissues such as stems and flowers [28] but not in roots or seeds, even after wounding [29,30]. Although phylogenetically restricted, Arabidopsides are not exclusive to *A. thaliana* and are also produced by other *Arabidopsis* species in much lower amounts and primarily under stress-related conditions, freeze thawing, and mechanical wounding [5,31,32]. Plant species producing OPDA-containing galactolipids include *A. arenosa*, *A. helleri*, *A. lyrata*, *A. petraea*, *A. suecica*, *A. lasiocarpa*, and *A. pedula* [5,32]. Arabidopsides were also reported to occur in some species from the genera of the Brassicaceae family (e.g., *Capsella rubella*, *Erucastrum canariense*, *Nasturtium officinale*, and *Neslia paniculata*) or not related to the Brassiceae (e.g., *Camelina microcarpa*, *Melissa officinalis*, and *Ipomoea tricolor)* [5,32].

## 5. Membrane-Localized Events Trigger the Biosynthesis of Arabidopsides

**Figure 1:**
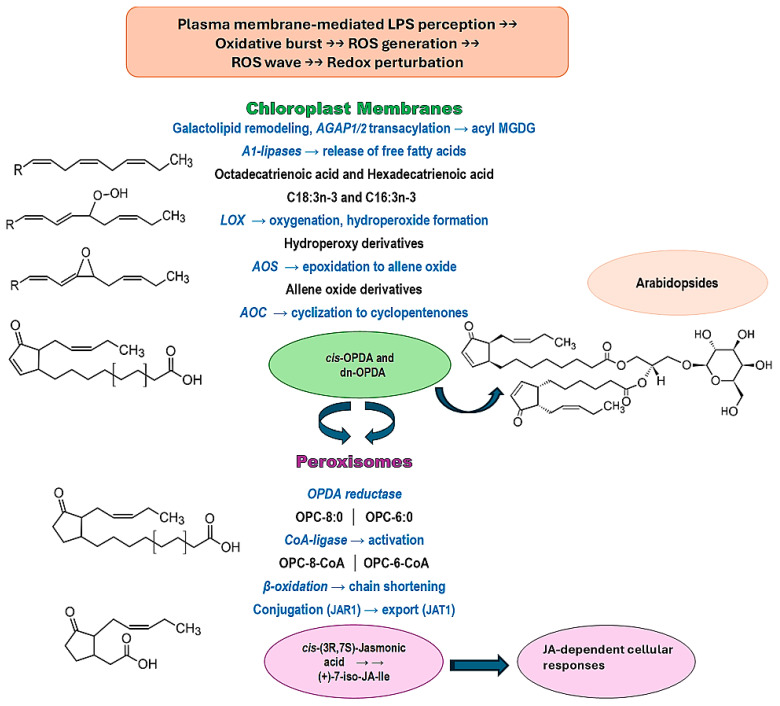
Outline of steps in cis-12-oxo-10,15-phytodienoic acid (OPDA) and jasmonic acid (JA) biosynthesis from C18:3n-3 and C16:3n-3 fatty acids and the relationship with Arabidopsides. Acylated galactolipid-associated phospholipase (AGAP) participates in membrane lipid remodeling. Release of free fatty acids by A_1_-lipases are followed by a cascade of enzyme-catalyzed steps indicated in blue—oxygenation (LOX, lipoxygenase), epoxidation (AOS, allene oxide synthase), and cyclization (AOC, allene oxide cyclase) to generate OPDA and dinor-OPDA. The square brackets in the structures of OPDA and OPC indicate the dinor derivatives where 2 methylene Carbon atoms were lost. Further enzymatic steps in the peroxisomes (reduction, ligation, and β-oxidation) via the OPC8/6 intermediates (OPC:8/6 = 3-oxo-2-pentenyl-cyclopentane-octa/hexanoic acid) produce cis-(3R,7S)-JA, with subsequent different metabolic conversions forming distinct JA-conjugates (e.g., JA-Ile) and other derivatives [37]. (JAR1 = JA-amino acid synthetase, JAT1 = JA transporter1).

The biosynthesis of Arabidopsides is relatively complex, involving a number of enzymes that may act on both lipid-bound fatty acids esterified in complex membrane lipids in plastids or potentially also free fatty acids (Figure 1) [5,15]. Following the perception of stress events at the PPM, extracellular reactive oxygen species (ROS) are generated by an NADPH oxidase (respiratory burst oxidase homolog D, RBOHD). A ‘ROS wave’ is responsible for the activation of systemic signaling pathways mediating ROS-dependent changes [33]. However, the routes of signal propagation and integration are unknown, and it is not certain how these signals convey specificity [33]. The effect of hydrogen peroxide and other reactive species is concentration- and context-dependent, acting as a signaling molecule at low concentrations and as an antimicrobial agent at high concentrations [34]. Early events following the perception of LPSs include an oxidative burst [20,35] that might be biphasic, with an initial burst located at the PPM, and a second, long-lasting burst associated with the chloroplast [35]. The activation of fatty acid oxidation cascades might follow the oxidative bursts [17,36].

Lipid remodeling involving post-synthetic structural modifications of membrane lipids is associated with plant responses to biotic and abiotic stresses [9,15,32]. These changes play crucial roles in generating bioactive molecules that function as second messengers in plant signal transduction pathways, particularly those related to pathogen defense and responses to wounding [9,15]. Acylated galactolipid-associated phospholipase (AGAP1 and the related AGAP2) is involved in lipid remodeling and primarily functions in the transacylation of galactolipids within chloroplast membranes. AGAP1 transfers a fatty acid from one monogalactosyl diacylglyceride (MGDG) to the galactose residue on the head group of another, producing an acyl-MGDG and a lysogalactolipid [15,38]. Acyl-MGDGs are believed to be part of the plant’s stress response mechanisms [32] and can serve as a reservoir for oxidized fatty acid derivatives, such as 12-oxo-phytodienoic acid (OPDA).

Figure 1 summarizes the generally accepted version of the steps leading to the synthesis of the OPDA [37]. Membrane lipid remodeling might include the release of free fatty acids from galactolipids through A1-type lipases. Subsequent oxidative steps yield hydroperoxides (e.g., hydroperoxy-hexadecatrienoic and hydroperoxy-octadecatrienoic acids), catalyzed by lipoxygenases at Carbon atom 13 (e.g., 13-LOX isozymes 2 and 4, with dioxygenase and hydroperoxidase activity) [39]. Alternatively, A1-type lipases can also hydrolyze oxygenated fatty acids from the membrane lipids and generate fatty acid hydroperoxides. These oxylipin-generating events may not be specific to the synthesis of Arabidopsides but contribute to the shared pool of available substrate also utilized for JA synthesis.

Subsequent downstream steps are catalyzed by allene oxide synthase (AOS) and allene oxide cyclase (AOC) [40]. These three chloroplastic enzymes were reported to operate as a multimeric protein complex (13-LOX, AOS, and AOC) or a defined metabolic shunt in *A. thaliana* [41]. The product of the combined enzyme activities is (cis-(+)-12-oxo-phytodienoic acid (OPDA)), a cyclopentenone. OPDA can thus be regarded as a cyclic derivative of 13(S)-hydroperoxolinolenic acid.

In addition, the shorter-chain homolog of OPDA, the 2,3-dinor-derivative (dn-OPDA, that has lost two methylene groups from its parent structure via a β-oxidation step), is also found [5,40,42]. Both the hexadecanoid and the octadecanoid pathways, as well as OPDA itself, can contribute to dn-OPDA synthesis. In *A. thaliana*, the major fraction of OPDA occurs esterified at the *sn* (stereospecific numbering)*-1* position of MGDGs [43]. Altogether, all steps in Arabidopside synthesis are enzyme-dependent and, apparently, all reactions can take place with substrates esterified to galactolipids [32,44].

Both OPDA and dn-OPDA are required for the synthesis of Arabidopsides (Section 6). The cyclopentenones contain an α,β-unsaturated carbonyl group and the electrophilic properties of this reactive center render cyclopentenones susceptible to conjugate addition reactions with various intracellular targets [45]. In accordance, OPDA may also be found conjugated to amino acids (Val, Phe, Ala, Glu, and Asp) [46].

Figure 1 also indicates the reactions occurring in peroxisomes to convert chloroplast-derived OPDA into cis-(3R,7S)-JA or (+)-iso-JA. JA can subsequently undergo different metabolic conversions in the cytosol to form distinct JA conjugates, e.g., the biologically active isoleucine conjugate (JA-Ile) that plays an important role in the induction and regulation of induced resistance responses in plants [37,40].

It is important to note that the metabolite flux through the central metabolic pathway as illustrated may be subject to stimulus-specific actions and substrate specificity of inducible isoenzymes against a tissue-specific background and downstream regulatory networks. Predictably, wounding of *A. thaliana* roots resulted in a decrease in preformed OPDA concomitant with an increase in JA and JA-Ile [29]. However, differential activities of the enzymes contributing to central metabolite pools and those withdrawing therefrom might contribute to tissue specificity by shifting the balance of flux towards OPDA (and Arabidopsides) vs. JA accumulation.

Intriguingly, based on work with disease resistance—and SA mutants of *A. thaliana*—it was reported that different signaling pathways (with different transcriptional and translational controls) support the synthesis of Arabidopsides during the hypersensitive response (HR, a specialized form of rapid, localized cell death) associated with defense, as compared to a wound response [47] (Section 7).

## 6. Structural Diversity of Arabidopsides

**Figure 2:**
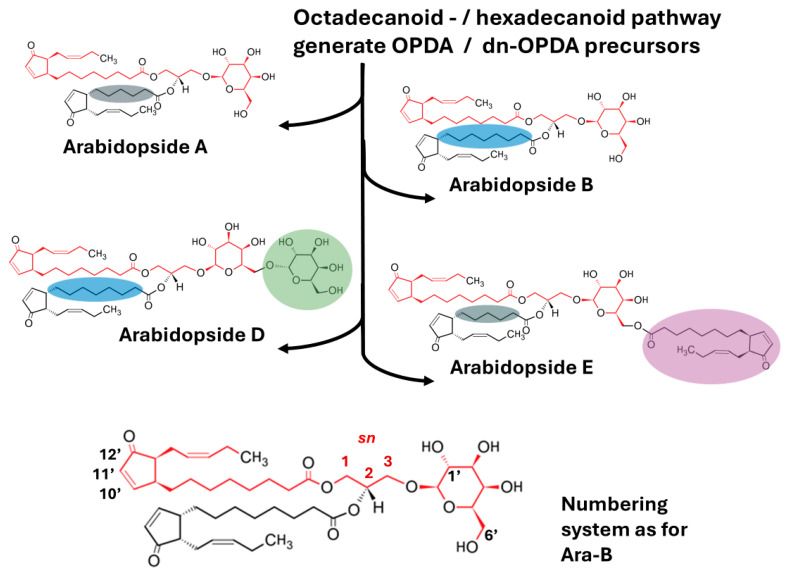
Arabidopside structures indicating the modular design supporting the chemical complexity and diversity. Arabidopsides differ in the number of polar vs. non-polar moieties and exhibit size heterogeneity resulting in sub-regions with different properties. Red = common structural features of all Arabidopsides, colored spheres = variable features, green = galactose, purple = OPDA, blue = OPDA chain, gray = dn-OPDA chain. The stereospecific numbering (*sn*) for glycerol, the 1′ and 6′ Carbon atoms of galactose, and the 10′, 11′, and 12′ Carbon atoms of the cyclopentenone ring of OPDA/dn-OPDA are indicated in the enlarged structure of Ara-B.

The glycerol-/galactolipids of *A. thaliana*, which is a (16:3) plant, contain either a (18:3) or (16:3) acyl group at the *sn-2* position. Chemically, Arabidopsides are mono- or digalactosyl glycerides, esterified to two fatty acid-derived oxylipins at the *sn-1* and *sn-2* positions of glycerol (Figure 2) to generate the corresponding mono- and digalactosyl diacylglycerides (MGDGs and DGDGs). The acylated oxylipins may thus consist of both OPDA and/or dn-OPDA as the cyclopentenones (Table 1) [4,48].

The chemical structures of Arabidopsides as oxidized galactolipids and the associated nomenclature have been summarized in detail [5,49,50]. Briefly, in *A. thaliana* these may contain OPDA or dn-OPDA bound to the glycerol backbone of the galactolipid and/or at the 6′ position of the galactosyl moiety. In the case of MGDG, as well as DGDG, OPDA is linked to the glycerol backbone at the *sn-1* position or at both the *sn-1* and *sn-2* positions as in Arabidopside B (Ara-B) and Ara-D. Similarly, when present, dn-OPDA is always esterified at the *sn-2* position to generate Ara-A (a MGDG) and Ara-C (a DGDG) respectively [4,48]. The *sn-3* carbon is attached to the 1′ position of a galactosyl residue via an ether bond. In addition to these MGDG and DGDG derivatives, acylated MGDG species like Ara-E [50] and Ara-G [5] have been identified. Here, a further OPDA molecule is esterified to the *sn-3* galactosyl moiety in addition to the OPDA at the *sn-1* and the OPDA/dn-OPDA at the *sn-2* position. Ara-G contains three OPDA moieties, while in the case of Ara-E, dn-OPDA is esterified at the *sn-2* position (Figure 2, Table 1). Phylogenetically conserved and inducible acylated galactolipid-associated phospholipases (AGAP1 and 2) located in the chloroplast envelope membranes were reported to catalyze the formation of head group-acylated galactolipids Ara-E and Ara-G [15,32,38].

Additional Arabidopsides containing hexadecatrienoic acid (HTA, 16:3) linked at the *sn-2* position and OPDA at the *sn-1* position (MGD-O) [43] or octadecatrienoic acid (ODTA,18:3) at the *sn-1* position and dn-OPDA at the *sn-2* position (Ara-F) were also identified [5,49]. As can be seen in Figure 2, the differences in the structures and numbers of OPDA, dn-OPDA, and galactose units can significantly alter physicochemical properties such as molecular size and hydrophobicity, potentially influencing the biological activities of individual Arabidopsides.

## 7. In Planta Function(s) of Arabidopsides

How the structural features of Arabidopsides relate to function is not yet fully understood [5]. As lipid-derived structures, it is possible that they may interact with other membrane lipids, modifying membrane organization and activating defense mechanisms. While the precise role(s) of Arabidopsides are still being investigated, these galactolipids are regarded as important signaling molecules involved in various developmental processes and as part of the plant’s response to stress, both abiotic and biotic [47]. In this context, they are increasingly recognized for their role as signaling agents [12,13] and possibly vital for defense mechanisms of plants containing Arabidopsides. Relatedly, Arabidopside formation was reported as dependent on intact JA signaling since levels of Arabidopsides were significantly reduced in the *coronatine insensitive1* (*coi1*) and *jasmonate resistant1 (jar1*) mutants compared to wild type following wounding or infection by virulent *Pseudomonas syringae* pv. *tomato* DC3000 [47].

Differential accumulation profiles of Arabidopsides due to wounding and virulent vs. avirulent pathogen exposure were interpreted as indicative of dissimilar operative signaling pathways in response to wounding vs. during the HR [47]. With regard to the latter, the synthesis of Arabidopsides seems to be linked to the HR and not to other types of defense response to pathogens.

The functional roles of Arabidopsides are inherently linked to that of OPDA, dn-OPDA, and JA. It has been proposed that Arabidopsides may fulfil dual functions, broadly categorized as contributing to antimicrobial defense towards potential pathogens and insects [51] and, secondly, as a phytohormone reservoir. Arabidopsides may serve as storage compounds or conjugates that, through the action of an acyl hydrolase, facilitate the slow release of OPDA and thus JA and related jasmonates. Other authors have argued for the rapid release of OPDA as precursor substrate for the synthesis of JA [40].

OPDA was hypothesized to act as a circuit breaker and/or a switch of plant growth and defense [52]. Although synthesized in different cellular compartments (chloroplast vs. peroxisome), OPDA is the primary precursor of JA. However, from a functional point of view, OPDA appears to fill physiological roles distinct from that of the bioactive JA-Ile [53,54,55] and may also act as a signaling compound in its own regard [52,55]. Here, OPDA induces gene expressions linked to specialized metabolism, stress responses, and detoxification [56]. Furthermore, OPDA acts in a cooperative manner with JA to optimize the expression of defense genes in *A. thaliana* [45]. The relationship between initial JA synthesized *de novo* as the primary response and JA formed upon release of OPDA from Arabidopsides as a secondary response is therefore still unresolved.

## 8. Analysis of Low Molecular Weight Metabolites

### 8.1. Metabolomics Tools and Approaches Offer Insight into Adaptive Responses

As the final recipient of biological information flow, the metabolome, in all its complexity, represents the phenotype of the plant [2]. Ultra-high-performance liquid chromatography coupled to a mass spectrometer with high definition, sensitivity, and resolution (UHPLC-MS) has emerged as a very suitable platform for metabolomics applied to phytochemical analysis [1,57]. Driven by improvements in analytical resolution and sensitivity, the scope of metabolomics has expanded, and the range of its applications in plant science research extended [2,3,58]. The use of chemometric tools along with metabolomics data provides a powerful method to capture the dynamic changes that occur in metabolites involved in resilience, tolerance, susceptibility, or disease resistance.

The investigation of low abundance metabolites that are not detected under steady state/homeostatic control conditions but only upon stress is associated with inherent difficulties [46] that can be addressed through metabolomics. Here, analysis of metabolomics data involved multivariate statistics (MVDA) for interpretation of LPS-induced metabolomic perturbations and reprogramming. MVDA methods clarify the underlying trends in complex data sets through the analysis of relationships between more than one characteristic at a time [3]. Two MVDA tools used to gain insights into the complex data sets were the principal component analysis (PCA) for the reduction in the dimensionality of the data to provide an intuitive global view of similarity and differences between and within samples and hierarchical clustering analysis (HiCA), which was also applied to explore group clustering and identify trends in the data. This was followed by the orthogonal partial least squares discriminant analysis (OPLS-DA) for predictive linear regression analysis and identification of potential biomarkers (or *m/z* ion features) that drive the group differences. In addition, the heatmap analysis was employed to compare the occurrence and abundance of selected metabolites between conditions and treatments [59,60].

Due to the different classes and unique features of specialized plant metabolites, annotation of the discriminant features can be complex and time-consuming [49,57]. To assist with annotation, newly developed computational procedures based on high-resolution mass spectrometric, artificial intelligence and machine learning, as well as in silico databases, opened up the possibility to search for unknown compounds, and they can pave a route forward [61,62].

### 8.2. Methodology Used for Probing LPS-Triggered Perturbations to the Metabolome

Metabolomic analyses of molecular responses triggered in *A. thaliana* leaves following exposure to intact LPSs have identified discriminant metabolites of the host response. The summarized data presented here was obtained from three different untargeted metabolomics studies [27,59,60]. The overarching strategy and rationale of the studies, as well as the common experimental procedures, are reported in detail in the individual reports.

An untargeted approach was followed using LPSs purified from three different bacteria: *Burkholderia cepacia* [27], *Xanthomonas campestris* pv. *campestris,* strain 8004, and *Pseudomonas syringae* pv. *tomato* DC3000 [59,60]. *B. cepacia* is a non-pathogen on *A. thaliana*, while the latter two are pathogenic. These LPSs represent different chemotypes of the standard LPS structure but differ in the type and number of repeat oligosaccharide units within the O-chain and the number and type of fatty acids within lipid A. The LPS from *B. cepacia* is tetra-/penta-acylated, while LPSs from *P. syringae* and *X. campestris* are penta-/hexa-acylated and hexa-acylated respectively. These structural characteristics influence the molecular shape of the LPSs, which in turn has been linked as a determinant of LPS biological activity [63,64].

*A. thaliana* leaves were pressure-infiltrated with LPS solutions, which was followed by sampling during the 0–24 h period and extraction of the metabolites in 80% aqueous methanol. The extracts were analyzed through UHPLC with gradient elution on a reverse phase column, able to separate mid-polar, as well as non-polar analytes. The data was acquired in both the positive and negative electrospray ionization modes. The mass detector was a high-definition, high-resolution quadrupole time-of-flight, accurate mass mass spectrometer (qTOF-MS, Waters Corporation, Milford, MA, USA). Raw data was analyzed using vendor-specific software (MassLynx XS^TM^ version 4.1, Waters Corporation, Manchester, UK). The processed data matrices were exported into the SIMCA-P software, version 14 (Sartorius, Umeå, Sweden), for statistical modeling, which included PCA and OPLS-DA. Detailed experimental procedures are reported in the cited papers [27,59,60].

The annotation of statistically significant features (*m/z* ions), compiled through OPLS-DA, was performed as tentative level 2 identifications according to the Metabolomics Standards Initiative guidelines [65]. The accurate mass, elemental composition, fragmentation patterns, and online database searches were used to facilitate assigning the correct chemical structure to each discriminant feature [27,59,60]. Online databases used for annotation were Metabolomics Workbench (https://www.metabolomicsworkbench.org), Lipid Maps (https://www.lipidmaps.org/), MetaCyc (https://metacyc.org/), ChemSpider (http://www.chemspider.com/), Pubchem (https://pubchem.ncbi.nlm.nih.gov/), and Dictionary of Natural Products (http://dnp.chemnetbase.com), as well as literature sources [27,28,49].

## 9. Arabidopsides as Discriminatory Biomarkers of LPS-Induced Defense Responses

The results indicate that the elicitation of *A. thaliana* with all three LPS chemotypes results in qualitatively similar alterations to the metabolome that are chemically distinct from that of non-treated leaves. This also suggests that possible differences in the perception mechanism(s) related to the molecular patterns found within the LPSs [27,59,60] may not play an important role in the sensing thereof as a ‘non-self’ entity. The fact that Arabidopsides have been reported to be responsive to both abiotic elicitation (e.g., physical wounding and chemical treatments) and biotic agents (e.g., gram-negative bacterial pathogens) prompted us to search the data sets for specific mass-associated fragments. Table 2 lists the discriminatory annotated metabolites corresponding to the metabolic pathway as illustrated in Figure 1. Relative peak intensities indicated a controlled, time-dependent, and transient increase in the responsive metabolites over the 0–24 h time period of investigation. LPS-elicited plants displayed a distinct and sustained elevation of these Arabidopsides (Ara-A, -B, -D, and -E), particularly evident at 12 and 24 h post treatment.

## 10. Discussion, Insights, and Perspectives

Plant responses to environmental cues can be considered as an information-processing network that describes how complex environmental signals are perceived, integrated, and processed [66]. Such information management acts like a ‘perceptron’ (or neural network) that processes inputs to produce adaptive outputs, crucial for survival as sessile organisms. Several perception units may operate in parallel, and signals are appropriately ‘weighted’ at subsequent levels to define an output transcriptome [66]. In the situation where a potential pathogenic threat is perceived, the perceptron view highlights the intricate molecular pathways linking MAMP sensing with hormone signaling and gene regulation, leading to flexible and appropriate adaptive changes, such as the activation of stimulus-specific defense responses.

Specialized metabolites are integrated components of metabolic networks that exhibit sensitivity to environmental triggers. Different functional classes of metabolites will exhibit different induction patterns or response kinetics. Following the gene transcript to protein to metabolite information flow, the majority of changes to the metabolome can be expected to become detectable from 12 h onwards. Due to the dynamic responsiveness of the metabolome, active metabolism occurs to return the system to a new homeostasis. Accordingly, the selected time points were 8, 12, and 24 h, which is in accordance with our previous work on defense metabolomes. The interpretation of the data in the framework of MAMP-triggered responses and plant–microbe interactions indicate that a clear cause-and-effect correlation (a relationship or pattern in time) exists between LPS treatments and the synthesis and accumulation of the annotated Arabidopsides.

Although much remains to be discovered about the perception of molecular patterns within LPS, the treatment of plant cells is associated with perception and signal generation. These events generate temporary molecular fingerprints in the cellular membranes (e.g., release of free polyunsaturated octadecanoic- and hexadecanoic acid, hydroxylation, hydroperoxidation, and epoxidation), followed by the initiation of a defense-related response focused on the generation of biologically active OPDA and JA/JA conjugates such as JA-Ileu (Table 2). The UHPLC-MS data clearly represents the activation of the octadecanoid pathway that is a central signaling pathway in plants that produce JA and other defensive molecules, or ‘octadecanoids’, from linolenic acid released from cellular membranes. In this case, due to the unique phytochemical features of specialized metabolism in *A. thaliana*, the flux through the pathway is channeled towards Arabidopsides. It should be emphasized that this might not be the situation in other plants, as the occurrence of Arabidopsides is phylogenetically limited, as mentioned in Section 4. In such cases, the activated octadecanoid pathway will generate JA and JA conjugates from the OPDA/dn-OPDA pools with different signatory biomarkers.

Previous findings have hinted that the basal composition of Arabidopsides is different from those that are formed following various stress treatments of *A. thaliana* [16]. Although the association of Arabidopsides with stress, wounding, and disease-induced responses is clear, interesting differential stress-specific signatures reported in the literature raise further questions. While wounding seemingly triggers the accumulation of high amounts of Arabidopsides A, B, E, and G, non-HR-dependent pathogen defense did not cause the accumulation of Arabidopsides [47]. In contrast, the HR seems to strongly favor the accumulation of Arabidopsides E and G, whereas Arabidopside A was found to decrease during early HR responses. Thus, the synthesis, and interconversion of Arabidopsides seem to be closely associated with cell death during the HR and not to other types of pathogen-induced defense responses. Moreover, the differences in Arabidopsides identified in plant leaves infiltrated with purified LPS from *P. syringae* and *X. campestris* (Ara-A, -B, and -E) vs. treatment of undifferentiated cell suspensions (where no Arabidopsides could be detected in cells or the growth medium) were also observed [59], underscoring the dynamic and condition-dependent nature of LPS-mediated metabolic reprogramming.

Regardless of the Arabidopsides’ signature, the intensity of the induced response needs to be regulated or attenuated. While considerable steps have been taken to gain deeper insights into the biological properties and biosynthesis of OPDA, the mechanisms by which plants regulate its levels are still not fully understood.

OPDA may serve as a metabolite hub, that acts as a central regulator of developmental vs. environmental signals (or biotic vs. abiotic interactions with the environment). As part of such an information processing network, the maintenance of OPDA homeostasis through reversible conjugation in the form of Arabidopsides will act to avoid toxicity from excessively high concentrations. The feedback inhibition of 13-LOXs by OPDA and JA has been proposed [39], but OPDA and JA levels can also be controlled through the conversion to inactive bio-conjugates. OPDA may also be found conjugated to certain amino acids, as well as glutathione. The latter is interesting since glutathione functions in redox sensing and signaling to respond to environmental change [67]. Although these conjugates were found in response to biotic and abiotic stresses in *A. thaliana* and other plants, they only displayed OPDA-related responses in a JA-Ile-dependent manner upon hydrolysis of the amino acid moiety and subsequent conversion to JA-Ile [46]. Such conjugation reactions may thus represent a metabolic mechanism to allow plants to adapt and respond to a wide range of physiological conditions that have in common the perturbation of OPDA and JA homeostasis.

Some evidence suggests that the synthesis of Arabidopsides acts independently of the initial rapid induction of JA signaling. This might be indicative of a positive feedback regulatory loop to amplify or intensify the response until a threshold or endpoint is reached. Relatedly, a possible explanation of the enhanced accumulation of Arabidopsides in response to elicitation by LPS is ‘priming’, which suggests that perception of LPS on its own is sufficient to trigger some aspects of protection but not the full spectrum of activated plant defense [22]. The primed or pre-conditioned state refers to a condition where a plant retains a ‘memory’ of a prior, milder stress or stimulus, which enables a quicker, stronger, and more efficient defense response in the face of subsequent, more severe biotic threats [58,59]. This primed memory has been described as a strategy of plant defense systems to sensitize, potentiate, or ‘immunize’ affected plants [68,69]. The plant’s immune system is thus primed without sustaining the metabolic expenses of launching a full defense unless an actual threat appears. In the context of plant resistance and defense, the priming effect can be particularly advantageous, as it enhances effective defensive capabilities of susceptible cultivars through metabolic reprogramming, which enables early induction and sustained activation of defense-related metabolic pathways. This is conceptually similar to a physiological state of ‘enhanced defensive capacity’ or ISR. Mechanistically, ISR is predominantly mediated through the JA and ethylene signaling pathways, which may function synergistically with SA-dependent signaling to reinforce host resistance against bacterial pathogens [68,69].

## 11. Emerging Significance and Future Perspectives

As previously mentioned, the data generated by untargeted metabolomics can point to novel connections and mechanisms and contribute to formulating new testable hypotheses for further, more targeted experiments. In addition, the integration of metabolomics data with other omics data can open the way to a more comprehensive, system-level understanding of biological processes and provide stronger mechanistic insights for the generated hypotheses.

One such topic of emerging significance and increasing importance is how the metabolism of fatty acids and lipids interface with the various types of plant signaling mechanisms induced in response to infection by microbial pathogens [12,13,17]. The integration of stress-derived signals that result in either specific or shared metabolic stress responses is still largely unknown. Research is ongoing to better understand the role of Arabidopsides (OPDA esters) and similar molecules (e.g., OPDA amides) in *A. thaliana*. Recent findings support the impression that plants utilize metabolic pathways at disposal to modulate OPDA and Arabidopside actions [46]. However, the mechanism by which OPDA derivatives are formed as well as their respective role(s) and function(s) still need to be better understood. Their exact contributory roles in plant–pathogen interactions and, specifically, their involvement in the signaling networks of innate immunity and defense require further investigation and elucidation.

Future research could also build on the present knowledge by exploring the structure–function relationships of bioactivities associated with Arabidopsides, which would provide further insights into their roles as specialized secondary metabolites and part of the defensive strategies employed by *A. thaliana.*

The suggested dual role for Arabidopsides, potentially delivering both immediate defense against microbial pathogens and serving as storage metabolites for impending JA release, requires further investigation and flux estimations of competing pathways and regulatory controls in the context of plant priming. There is still much to uncover in order to understand the potentiation of plant defense mechanisms, since the molecular events fundamental to the priming phenomenon may vary or overlap depending on the initial stimulus, the inherent metabolic capabilities of the plant, and the secondary triggers.

In this context, novel metabolomic insights indicate that reprogramming of metabolomes is a crucial adaptive process in coping with stress-related perturbations. When metabolic changes last beyond the recovery from stress events, it establishes an altered state of metabolism or metabolic imprint. Metabolic imprints may thus serve as a memory that stores and processes information [70,71]. In this context, metabolic reprogramming, linked to priming, can be viewed as an imprint that reflects the integration of preceding memories of environment-derived signals and functions to prepare the plant for future events [71], i.e., to act as a central component of plant priming.

## Figures and Tables

**Table 1:** Modular compositions of Arabidopsides A to G and associated physicochemical properties of importance to mass spectrometric (MS) analyses.

Arabidopside	Modular Composition *			PubChem CID	Exact Mass	Empirical Formula
	Gal	OPDA	dn-OPDA			
Ara-A	1	1	1	163194994	774.4554	C_43_H_66_O_12_
Ara-B	1	2	-	162911818	802.4867	C_45_H_70_O_12_
Ara-C	2	1	1	163186618	936.5083	C_49_H_76_O_17_
Ara-D	2	2	-	162955405	964.5396	C_51_H_80_O_17_
Ara-E	1	2	1	16747780	1048.6487	C_61_H_92_O_14_
Ara-F	1	-	1	171119307	760.4762	C_43_H_68_O_11_
Ara-G	1	3	-	102419651	1076.6800	C_63_H_96_O_14_

* The number of constituent OPDA (cis-12-oxo-10,15-phytodienoic acid), dn-OPDA, and galactose (Gal) residues as structural modules attached to the glycerol backbone of the respective Arabidopsides. CID = compound identifier.

**Table 2:** Summary of the occurrence of Arabidopsides and related analytes in methanolic extracts of *A. thaliana* leaves, annotated as discriminant metabolites in response to elicitation with lipopolysaccharides purified from *Burkholderia cepacia* [27], *Xanthomonas campestris* pv. *campestris*, strain 8004 [59] and *Pseudomonas syringae* pv. *tomato* [60]. The presence of oxylipins as precursors and the co-occurrence of OPDA and JA and derivatives thereof, are also tabled.

Metabolites *	Empirical Formula	Adduct	*m/z*
**Octadecanoic–and hexadecanoic acids, hydroxy and hydroperoxyl derivatives**			
Octadeca-9,11,15-trienoic acid	C_18_H_30_O_2_	[M+H]**^+^**	279.2319
17-Hydroxy-octadecatrienoic acid	C_18_H_30_O_3_	[M+H]**^+^**	295.2200
12,13-Epoxy-octadecatrienoic acid	C_18_H_28_O_3_	[M−H]^−^	292.2029
9,10-Epoxy-octadecatrienoic acid	C_18_H_28_O_3_	[M+H+K]**^+^**	331.1650
7,8-Dihydroxy-9,12-octadecadienoic acid	C_18_H_32_O_4_	[M−H]^−^	311.2210
9,12,13-Trihydroxy-octadec-10-enoic acid	C_18_H_34_O_5_	[M−H]^−^	329.2321
9,12,13-Trihydroxy-10,15-octadecadienoic acid	C_18_H_32_O_5_	[M−H]^−^	327.2161
13-Hydroperoxy-9,11,15 octadecatrienoic acid	C_18_H_30_O_4_	[M−H]^−^	309.2063
9-Oxo-octadecenoic acid	C_18_H_30_O_3_	[M−H]^−^	293.2085
9,10-Dihydroxy-8-oxo-12-octadecenoic acid	C_18_H_32_O_5_	[M−H]^−^	327.2146
15,16-Dihydroxy-9,12-octadecenoic acid	C_18_H_32_O_4_	[M−H]^−^	311.2183
Hexadecanoic acid	C_16_H_32_O_2_	[M+K−H]^−^	293.2082
9-Hexadecenoic acid	C_16_H_30_O_2_	[M−H]^−^	253.0900
16-Hydroxyhexadecanoic acid	C_16_H_32_O_3_	[M−H]^−^	271.2243
10,16-Dihydroxy-hexadecanoic acid	C_16_H_32_O_4_	[M−H]^−^	287.2201
**Oxo-phytodienoic acid (OPDA) and derivatives**			
12-oxo-phytodienoic acid (12-OPDA)	C_18_ H_28_O_3_	[M−H]^−^	291.1930
12-OPDA	C_18_H_28_O_3_	[M+H]**^+^**	293.2138
Dinor-12-oxo-phytodienoic acid	C_16_H_24_O_3_	[M−H]^−^	263.1631
**Arabidopside precursors**			
3′-O-Linolenoylglyceryl 6-O-digalactosyl monoglyceride	C_33_H_56_O_14_	[M−H+FA]^−^	721.3663
*sn-2*-O-(dinor-OPDA)-monogalactosyl monoglyceride	C_25_H_40_O_10_	[M−H+FA]^−^	545.2611
*sn-2*-O-(dinor-OPDA)-digalactosyl monoglyceride	C_31_H_50_O_15_	[M−H+FA]^−^	707.3175
*sn-1*-O-(OPDA)-digalactosyl monoglyceride	C_33_H_54_O_15_	[M−H+FA]^−^	735.3514
**Arabidopsides**			
Ara-A	C_43_H_66_O_12_	[M+FA−H]^−^	819.4540
Ara-A	C_43_H_66_O_12_	[M+H]**^+^**	775.4630
Ara-B	C_45_H_70_O_12_	[M+FA−H]^−^	847.4880
Ara-B	C_45_H_70_O_12_	[M+Na+H]**^+^**	825.4690
Ara-D	C_51_H_80_O_17_	[M−H+FA]^−^	1009.500
Ara-E	C_61_H_92_O_14_	[M+H]**^+^**	1049.5000
**Jasmonic acid (JA) and derivatives**			
Jasmonic acid	C_12_H_18_O_3_	[M+H]^+^	211.1303
Jasmonoyl-L-isoleucine	C_18_H_28_NO_4_	[M−H]^−^	322.2083
9,10-Dihydrohydroxy-jasmonic acid sulphate	C_12_H_20_O_7_S	[M−H]^−^	307.0833
(9R,13R)-1a,1b-Dihomo-jasmonic acid	C_14_H_22_O_3_	[M+Na]**^+^**	261.1461

* Annotations were according to the Metabolomics Standards Initiative, level 2.

## Data Availability

All relevant data is contained in the manuscript. No new data is presented.

## Abbreviations

The following abbreviations were used in this manuscript:

AGAP	acylated galactolipid-associated phospholipase
AOC	allene oxide cyclase
AOS	allene oxide synthase
Ara-X	arabidopside (A/B/C/D/E/F)
COS	core oligosaccharide
DGDG	digalactosyl diacylglyceride
dn-OPDA	dinor-oxo-phytodienoic acid
Gal	galactose/galactosyl
HiCA	hierarchical cluster analysis
HR	hypersensitive response
ISR	induced systemic resistance
JA	jasmonic acid
JA-Ile	JA isoleucine conjugate
JAR	JA-amino acid synthetase
JAT	jasmonic acid transporter
LOX	lipoxygenase
LPS(s)	lipopolysaccharide(s)
MAMP	microbe-associated molecular pattern
MGDG	monogalactosyl diacylglyceride
MS	mass spectrometry
MVDA	multi-variate data analysis
OPS	O-polysaccharide
OPLS-DA	orthogonal partial least squares discriminant analysis
OPDA	oxo-phytodienoic acid
PCA	principal components analysis
PR	pathogenesis-related
PRR	pattern recognition receptor
PPM	plant plasma membrance
RLK	receptor-like kinase
ROS	reactive oxygen species
SA	salicylic acid
UHPLC	ultra-high performance liquid chromatography
